# P053 When the aorta speaks before the spine: a case of extensive aortitis leading to the underlying diagnosis of HLA-B27-positive axial spondyloarthritis

**DOI:** 10.1093/rap/rkag113.083

**Published:** 2026-11-03

**Authors:** Abdul Hai Abdul Hai, Md Shahriar Kabir, Hazem Youssef

**Affiliations:** Department of Rheumatology, Aberdeen Royal Infirmary, NHS Grampian, Aberdeen, United Kingdom; Department of Rheumatology, Aberdeen Royal Infirmary, NHS Grampian, Aberdeen, United Kingdom; Department of Rheumatology, Aberdeen Royal Infirmary, NHS Grampian, Aberdeen, United Kingdom

## Abstract

**Introduction:**

Not all cases of large vessel vasculitis represent primary vasculitic disease. Identifying secondary causes of aortitis can be diagnostically challenging, particularly when vascular inflammation occurs in the absence of classical symptoms of the underlying condition. We report an unusual case of extensive thoraco-abdominal aortitis that ultimately led to the diagnosis of HLA-B27-positive axial spondyloarthritis after more than a decade of unexplained symptoms. This case demonstrates how multimodality imaging, multidisciplinary collaboration and careful re-evaluation of previous imaging can uncover occult inflammatory disease and fundamentally alter long-term management.

**Case description:**

A 64-year-old woman was referred to Rheumatology after a CT scan, performed to investigate persistent right upper quadrant pain, chest discomfort and a small pleural effusion, demonstrated extensive circumferential mural thickening involving the ascending, arch, descending thoracic and abdominal aorta.

Retrospective review revealed a history spanning more than a decade of unexplained chest symptoms, including presumed viral pericarditis, recurrent interscapular pain with paraesthesia, fatigue, proximal limb aching and possible exertional claudication, but without constitutional symptoms or clinically significant inflammatory back pain. Examination demonstrated symmetrical peripheral pulses and no evidence of inflammatory arthritis. Serial inflammatory markers were predominantly normal, with only occasional modest CRP elevation (peak 30 mg/L), despite extensive aortic involvement. ANA, ANCA, rheumatoid factor, anti-CCP antibodies and IgG4 were negative.

PET-CT demonstrated patchy FDG uptake involving the ascending, arch and descending thoracic aorta. CT angiography excluded chronic dissection and confirmed multifocal mural thickening consistent with inflammatory aortitis. Following multidisciplinary vasculitis review, she was treated with high-dose prednisolone followed by mycophenolate mofetil as a steroid-sparing agent, resulting in marked improvement in fatigue, limb symptoms, and normalisation of CRP and plasma viscosity.

Despite clinical improvement, surveillance MR angiography demonstrated persistent vessel wall oedema and progressive ascending aortic dilatation. Re-evaluation at the radiology MDT identified previously overlooked bilateral sacroiliac erosive changes on earlier CT imaging. Dedicated MRI of the sacroiliac joints confirmed chronic bilateral sacroiliitis without active inflammation, and HLA-B27 testing was positive. The diagnosis was subsequently revised to HLA-B27-positive axial spondyloarthritis with secondary aortitis.

Management was redirected towards TNF inhibitor therapy while mycophenolate was continued during the treatment transition. Follow-up imaging has demonstrated stable vascular disease, enabling successful glucocorticoid withdrawal with sustained clinical remission.

**Discussion:**

This case illustrates the diagnostic complexity of apparently idiopathic aortitis. Extensive aortic involvement on CT, PET-CT and MR angiography initially favoured primary large vessel vasculitis. However, the absence of constitutional features, persistently normal or only minimally elevated inflammatory markers, and a broad differential diagnosis, including chronic aortic dissection, prompted continued diagnostic re-evaluation. Sequential multimodality imaging, interpreted through repeated multidisciplinary discussion, proved pivotal in establishing the underlying diagnosis.

Although cardiovascular involvement is a recognised extra-articular manifestation of axial spondyloarthritis, clinically significant aortitis is uncommon and typically develops after years of established axial disease. In contrast, our patient presented with extensive thoraco-abdominal aortitis more than a decade before recognition of HLA-B27-positive axial spondyloarthritis, despite minimal spinal symptoms and persistently normal inflammatory markers. While her recurrent unexplained interscapular and chest pain could have been attributable to the aortitis itself, it is also possible that these episodes reflected intermittent thoracic spine or costovertebral joint inflammation as part of her underlying axial spondyloarthritis. The diagnosis only became apparent after re-evaluation of previous cross-sectional imaging identified previously overlooked sacroiliac abnormalities, prompting dedicated MRI and HLA-B27 testing.

Recognition of the underlying diagnosis fundamentally altered long-term management. Initial treatment with corticosteroids and mycophenolate was appropriate for presumed isolated aortitis and resulted in clinical and biochemical improvement. However, confirmation of HLA-B27-positive axial spondyloarthritis prompted transition to TNF inhibitor therapy, targeting the underlying inflammatory disease rather than presumed primary large vessel vasculitis.

This case highlights the importance of considering secondary causes of apparently idiopathic aortitis. Careful review of existing cross-sectional imaging may identify occult axial spondyloarthritis, even in the absence of classical inflammatory back pain, facilitating disease-specific therapy and potentially improving long-term vascular outcomes.

**Key learning points:**

• Not all cases of large vessel vasculitis represent primary vasculitic disease; secondary causes of aortitis should be considered when the clinical presentation is atypical.

• Aortitis may be the presenting manifestation of previously unrecognised HLA-B27-positive axial spondyloarthritis, even in the absence of clinically significant inflammatory back pain.

• Sequential multimodality imaging and careful re-evaluation of previous cross-sectional imaging can be pivotal in establishing the underlying diagnosis.

• Incidental sacroiliac abnormalities on cross-sectional imaging should prompt dedicated assessment for underlying axial spondyloarthritis in patients with unexplained aortitis.

• Multidisciplinary collaboration is essential in diagnostically challenging aortitis, facilitating accurate diagnosis and guiding disease-specific long-term management

